# Review of the Campaign to Prevent Falls in Construction

**DOI:** 10.3389/fpubh.2017.00275

**Published:** 2017-10-30

**Authors:** Kevin O’Donnell

**Affiliations:** ^1^Boston University, North Andover, MA, United States

**Keywords:** occupational health, health education, intervention, construction industry, promotion focus

## Abstract

Roofing is one of the most dangerous activities in the construction industry according to the US Bureau of Labor Statistics. Although injuries are manifold in this industry element, the vast majority of them occur as a result of falls from elevation (1, 2). These events lead to physical injury, fatalities, and financial burdens to the individual injured, their families, the employer, and the construction market as a whole (3, 4). In order to reduce construction worker falls, Occupational Health and Safety Administration launched the nationwide Campaign to Prevent Falls in Construction on April 26, 2012 (5). The campaign applied several learning theories that are utilized and proven effective in public health interventions. However, the initiative fails to address a key subpopulation. Further critical assessment of this campaign is now needed to evaluate overall effectiveness.

## Needs Assessment

Roofing is one of the most dangerous activities in the construction industry. According to the Bureau of Labor Statistics, workers in the roofing industry are over three times more likely to experience fatal occupational injuries when compared to construction workers in general (1, 2). Falls are the leading producer of roofing construction workplace fatalities, accounting for 76 fatalities between 2003 and 2009 (3). Falls not only injure construction workers but also increase financial burdens for their families, employers, and society. Between 2005 and 2007, 38 states that participate in the National Council on Compensation Insurance reported that falls from elevations cost insured roofers $54 million per year, approximately $106,000 per injured roofer, which is twice the average expenditure for other occupation-based elevation falls (4).

Within the profession of roofing construction, researchers have found that residential roofers, roofers who work on residential structures, are more likely to experience fall injuries than commercial roofers, those roofers who work on commercial buildings and properties (5). Residential roofers are less likely to be provided with fall protection devices and their employers generally do not enforce or require the use of safety equipment (6). Unlike their commercial counterparts, residential roofers are more likely to work for small employers, fewer than 10 employees (6). Kaskutas et al. found that use of personal fall arrest systems and monitoring of unprotected floor openings are rare among small establishments in construction (7). And, as highlighted by Olbina et al., small construction firms, in specific specialty contractors, are challenged to provide employees with a safe work environment (8). Further, the most at risk are generally Hispanic individuals on residential roofing worksites (9). Overall, these studies found that lack of fall protection programs, poor enforcement, and application of fall protection device use, residential roofing construction work, and small construction companies, all increased the risk of experiencing fall injuries within the roofing profession (9).

## Intervention

In order to reduce construction worker falls, Occupational Health and Safety Administration (OSHA) launched the nationwide Campaign to Prevent Falls in Construction (CPFC) on April 26, 2012. CPFC is developed from an initiative from OSHA Region 1 Area Office in Andover, MA, USA (10). OSHA and the National Institute for Occupational Safety and Health, the National Occupational Research Agenda—Construction Sector, and the Center to Protect Worker Rights (CPWR) developed this endeavor (11).

Campaign to Prevent Falls in Construction has three key messages: (1) working at heights should be planned so it is safe, (2) contractors must provide the proper equipment for working at heights and workers must use that equipment, and (3) workers need training in equipment use and how to work safely in the environment (11). In order to successfully adapt and influence individual roofer behaviors, CPFC posters, fact sheets, and training materials, such as videos, are provided to roofing companies (11). The campaign attempts to educate roofers and promote workplace safety by raising awareness of environmental hazards and by teaching methods of danger prevention; it endeavors to empower the individual to take actions and personal responsibility regarding the prevention of falls (11).

The hiring norms for the roofing industry are to obtain employees through temporary agencies, employee references, and parking lots, or other known locations where day-laborers wait for the opportunity to hire onto a job (10). Many of these laborers do not speak English well and are foreign-born (10). CPFC provides bilingual materials and OSHA offers compliance assistance meetings in Spanish, Portuguese, and English (11).

The campaign focuses on altering individual-based behaviors and as such, the CPFC intervention primarily applies the constructs of the Health Belief Model (HBM) and McGuire’s Psychological Motives (12). The HBM is derived from psychological and behavioral theories that ultimately claim the two components of health-related behavior are individuals want to avoid illness or hazards and if exposed want to do something to remedy that (13). According to the HBM, an individual’s course of action often depends upon the person’s perceptions of the benefits and barriers related to health behavior (13). CPFC applies several of the HBM constructs, such as perceived susceptibility, perceived severity, perceived benefits, and the cue to action, to its intervention.

Perceived susceptibility is defined as an individual’s subjective perception of the risk of acquiring an illness or disease (13). CPFC utilizes this construct by recounting personal experiences of both injured roofers and family members of injured or killed roofers as suggested by contagion theory and tension-reduction theory, making use of credible respectable spokespeople and the tactic of fear, respectively (13, 14).

Perceived severity, an individual’s feelings on the seriousness of contracting an illness or disease (14), is another element that CPFC utilizes by integrating the personal accounts that describe the physical and economic consequences of falling that an individual may bear (13). The perceived benefits element is an individual’s perception of the effectiveness of various actions available to reduce the threat (13). The campaign utilizes its flyers, posters, and educational videos to this end (11). It also utilizes individual accounts in an attempt to educate roofers about the use of proper safety equipment and safety procedures, which both lead to positive health outcomes by offering a positive role model to affirm positive social norms (11, 13, 14).

Finally, CPFC incorporates cues to action or the internal or external stimulus required to trigger the decision-making process that can lead to the acceptance of the recommended health intervention (11, 13). The campaign’s candid personal accounts are fear tactics and endeavor to prevent falls out of fear of consequences. CPFC empowers employees to seek necessary equipment, training, or avoid risky situations entirely (11, 13).

Campaign to Prevent Falls in Construction, however, neglects economic realities among roofers and focuses too strongly on personal responsibility. In other words, the campaign’s emphasis on personal empowerment can be destructive in situations where the ultimate cause of the roofer’s injury is circumstances beyond the workers’ control. A review of campaign materials reveals that they focus on the individual’s decision to use personal protective equipment in order to prevent the risk of falling, but the reality for many of these workers is that they do not have access to the equipment. Further, in many circumstances, the individuals have no control over those interventions other than to choose to work the job or not. The campaign fails to incorporate the influence of social norms and peer influences, as suggested by the social norms theory, on a roofer’s overall decision to work a job (14). It is especially important to consider these influences when working with younger, non-English speaking, foreign born people, which are the basis of socially stratified groups where norms and peer pressures are prevalent and often drive behavior (5). These two groups comprise the majority of the employees most affected by falls in the roofing industry and should be targeted by the campaign (5). These workers often are so desperate for a wage that they are willing to sacrifice their own safety and well-being in order to earn one.

In preventive health behaviors, early studies showed that perceived susceptibility, benefits, and barriers were consistently associated with the desired health behavior (14) while perceived severity was less often related with the desired health behavior (14). Individual HBM constructs are useful, depending on the health outcome of interest, but for the most effective use of the HBM, the campaign should integrate other theory-based constructs that account for the environmental context and suggest strategies for change.

Campaign to Prevent Falls in Construction does not properly focus on the group most at risk: non-commercial, non-Caucasian individuals who are more often day-workers than full time employees (11). CPFC testimonial videos either show seriously injured white men who are union employees and have fallen at commercial sites or show family members directly affected by a large commercial construction site fall (11). These videos do not represent the most at risk roofer population: Hispanic residential roof workers an essential element as presented in communication theory (15).

Since posters and handout materials clearly indicate that they are from OSHA, a government agent, and, since the targeted audience most at risk is largely comprised of day-workers who may in fact be illegal aliens or hail from foreign cultures who distrust government agencies, the audience may be fearful of the recommendations (10). Research indicates that the focus of the intervention should be for smaller employers (5, 16). CPFC is designed for a company that has a preexisting safety department and program, which are less likely to exist in small-scale roofing companies.

Because OSHA is primarily a law enforcement entity, it has limited outreach capabilities (10). CPFC focuses on the individual, and endeavors to tell people not to fall and to use proper safety equipment. However, most of these employers will not have, or even have access to, fall prevention equipment. Therefore, alternatively, a program that shifts the focus to homeowners, while not under the purview of OSHA, would be within the bailiwick of a group such as the CPWR. Using the HBM, the program could be modified to address the elements of perceived susceptibility by informing the homeowner that people do fall from roofs while performing services and can die. This information could not only appeal to the homeowner’s sense of personal responsibility but also liability because, if the contractor was uninsured, the homeowner is financially responsible for accidents on their property (10). Any future outreach through CPFC should make use of this HBM tool.

The CPFC attempts to make use of the social norms theory to redefine the operational norms of the construction industry by attempting to influence behavior by applying contagion theory and using positive role model examples to redefine what the social norms should be (17). They want to make it popular to utilize fall arrest equipment and safe work practices. However, the campaign is not successful in this regard, because it does not address the most vulnerable population according to literature (5).

It is further recommended that the program provide materials that focus more toward the vulnerable population: Hispanic residential roofers. Materials, although available in Spanish, do not address the specific needs of the audience most at risk and the materials don’t address the culture of construction that research indicates are the most vulnerable (11). Current video training materials do not address population most at risk or specify the work environment and, therefore, should be modified to show Hispanic individuals on residential roofing worksites (11).

## Evaluation

In the fiscal year of 2013, there have been no fatalities related to falls from roofs. Last year, there were eleven fall fatalities in the Andover district (10). Compliance assistance meetings are well attended by contractors and employees continue to demonstrate an interest in the education (10). Full effectiveness of the program is yet to be determined as no formal evaluation of this project is underway, which questions the campaign’s overall effectiveness. Efficacy would be definitively proven by an interventional clinical trial. The campaign has been in existence for 1 year and is ripe for assessment, modifications, and recommendations outlined.

## About the Author

Kevin O’Donnell, MS, MS Env. Eng., MS Construction Project Management, MPH former research assistant on the University of Massachusetts-Lowell Department of Work Environment’s Construction Occupational Health Project with over 20 years of experience as a journeyman electrician, safety compliance officer, and site project manager in residential, commercial, and heavy industrial construction; familiar with the cultural mores, social norms, and practices in the construction industry; has written several unpublished works concerning economic impacts on construction safety and has conducted numerous focus and participatory research groups as well as academic lectures on ergonomics, safety, and operations in the construction industry.

## Author Contributions

KO researched and wrote this piece.

## Conflict of Interest Statement

The author declares that the research was conducted in the absence of any commercial or financial relationships that could be construed as a potential conflict of interest.
